# Vitamin D and Breast Cancer: Mechanistic Update

**DOI:** 10.1002/jbm4.10582

**Published:** 2021-12-10

**Authors:** JoEllen Welsh

**Affiliations:** ^1^ Department of Environmental Health Sciences SUNY Albany Cancer Research Center Rensselaer NY USA

**Keywords:** CANCER, CELL/TISSUE SIGNALING—ENDOCRINE PATHWAYS, PTH/VIT D/FGF23, NUTRITION

## Abstract

The presence of the vitamin D receptor (VDR) in mammary gland and breast cancer has long been recognized, and multiple preclinical studies have demonstrated that its ligand, 1,25‐dihydroxyvitamin D (1,25D), modulates normal mammary gland development and inhibits growth of breast tumors in animal models. Vitamin D deficiency is common in breast cancer patients, and some evidence suggests that low vitamin D status enhances the risk for disease development or progression. Although many 1,25D‐responsive targets in normal mammary cells and in breast cancers have been identified, validation of specific targets that regulate cell cycle, apoptosis, autophagy, and differentiation, particularly in vivo, has been challenging. Model systems of carcinogenesis have provided evidence that both VDR expression and 1,25D actions change with transformation, but clinical data regarding vitamin D responsiveness of established tumors is limited and inconclusive. Because breast cancer is heterogeneous, the relevant VDR targets and potential sensitivity to vitamin D repletion or supplementation will likely differ between patient populations. Detailed analysis of VDR actions in specific molecular subtypes of the disease will be necessary to clarify the conflicting data. Genomic, proteomic, and metabolomic analyses of in vitro and in vivo model systems are also warranted to comprehensively understand the network of vitamin D–regulated pathways in the context of breast cancer heterogeneity. This review provides an update on recent studies spanning the spectrum of mechanistic (cell/molecular), preclinical (animal models), and translational work on the role of vitamin D in breast cancer. © 2021 The Author. *JBMR Plus* published by Wiley Periodicals LLC on behalf of American Society for Bone and Mineral Research.

## Overview: Breast Cancer Cells as Targets for Vitamin D Actions

### Detection of VDR in normal and cancerous breast cells

In 1979, the discovery of the vitamin D receptor (VDR) in cultured cells derived from human breast cancer^(^ ^1^ ^)^ triggered intense interest in the potential relationship between the vitamin D endocrine system and breast carcinogenesis, which continues to this day. At that time, competitive binding assays, sucrose density gradients, and Scatchard analyses were used to demonstrate specific, high‐affinity, low‐capacity binding of tritiated 1,25‐dihydroxyvitamin D (1,25D) in cytosolic or nuclear extracts of cultured cells. This was the first such evidence of VDR in any cancer cell and generated particular interest because of the propensity of breast cancer to metastasize to bone and induce hypercalcemia. In a follow‐up study, 1,25D binding sites were detected in breast and lymph node tissue from 7 of 10 patients with breast cancer and in patients with benign neoplasms.^(^ ^2^ ^)^ Cloning of the hVDR cDNA and generation of anti‐VDR monoclonal antibodies eventually confirmed that the majority of human breast tumors express VDR mRNA and protein, although the relationship between its expression and clinical outcomes remains unclear as discussed below.^(^ ^3^ ^)^

### Metabolism of 1,25D in breast cancer cells in vitro

An additional level of complexity of vitamin D action in cancer cells is imposed by the presence of vitamin D metabolic enzymes. Both CYP27B1 (the gene that encodes the 25‐hydroxyvitamin D 1α‐hydroxylase) and CYP24A1 (the gene that encodes the 25‐hydroxyvitamin D 24‐hydroxylase) have been detected in normal breast epithelial cells, in breast cancer cell lines and in patient derived tissue.^(^ ^4^, ^5^, ^6^ ^)^ The presence of both VDR and CYP27B1 in normal human breast epithelial cells confers sensitivity to growth inhibition by 25‐hydroxyvitamin D (25D) at physiological concentrations.^(^ ^4^, ^7^ ^)^ Furthermore, mammary epithelial cells express megalin and cubilin and internalize 25D bound to its serum binding protein (D binding protein [DBP], encoded by the GC gene).^(^ ^7^ ^)^ Comparison of normal breast epithelial cells to oncogene‐transformed derivatives indicated that both VDR and CYP27B1 were downregulated during in vitro transformation, leading to decreased sensitivity to both 25D and 1,25D.^(^ ^8^ ^)^ Conversely, upregulation of CYP24A1 in breast cancer cells would be predicted to reduce cellular sensitivity to 1,25D.

In addition to VDR, several other nuclear receptors such as the estrogen‐related receptor (ERR), the progesterone receptor (PgR), the pregnane X receptor (PXR), and the constitutive androstane receptor (CAR) have been shown to influence CYP24A1 expression and/or activity.^(^ ^9^, ^10^, ^11^, ^12^ ^)^ A recent study demonstrated that alcohol intake enhanced vitamin D catabolism in tumor‐bearing mice.^(^ ^13^ ^)^ It has also been reported that CYP24A1 was translationally upregulated in breast cancer cells treated with supernatants of activated monocyte‐derived macrophages (to mimic tumor inflammation). This study demonstrated the presence of an IRES element within the 5'UTR of CYP24A1 mRNA that was activated by the inflammatory milieu in a PI3K‐dependent manner, potentially linking a common oncogenic pathway in human breast cancer (AKT‐PI3K) with activation of vitamin D catabolism.^(^ ^14^ ^)^ Although these and other studies have identified several mechanistic pathways that alter vitamin D metabolism in vitro, additional factors likely contribute to deregulation of vitamin D metabolic enzymes in human breast tumors as discussed below.

### General anticancer effects of VDR agonists in breast cancer cell lines and animal models

Activation and subsequent nuclear translocation of VDR upon ligand binding suggested the likelihood that 1,25D availability could affect the biology of breast cancer cells. Indeed, it was rapidly established that 1,25D and a variety of synthetic VDR agonists exerted anticancer effects (including cell cycle arrest,^(^ ^15^ ^)^ apoptosis,^(^ ^16^ ^)^ and differentiation^(^ ^17^ ^)^) in VDR‐positive breast cancer cells in vitro as well as in some animal tumor models.^(^ ^18^, ^19^, ^20^ ^)^ Other effects of 1,25D that were demonstrated in various breast cancer model systems included blockade of epithelial‐mesenchymal transition (EMT),^(^ ^21^, ^22^ ^)^ invasion,^(^ ^19^ ^)^ metastasis,^23^, ^24^, ^25^ ^)^ and energy metabolism,^25^, ^26^, ^27^, ^28^, ^29^, ^30^ ^)^ as well as induction of autophagy.^(^ ^31^, ^32^, ^33^, ^34^, ^35^ ^)^ However, the specific mechanisms and pathways that link the 1,25D‐VDR complex to the observed biological effects remain elusive and appear to be highly cell type specific based on mechanistic studies and genomic profiling as described below.^(^ ^36^, ^37^, ^38^, ^39^, ^40^, ^41^, ^42^ ^)^

It should be noted that the concentrations of 1,25D utilized in vitro (typically 100 nM) have been chosen to maximally activate VDR, but are well above the physiological levels associated with dietary intake or epidermal synthesis. Early studies of 1,25D treatment in animal models of breast cancer rarely achieved anticancer effects without induction of severe hypercalcemia, generating pharmaceutical interest in the development of synthetic vitamin D analogs with activity profiles that favored growth inhibition over calcemic effects. Many of these analogs were effective at picomolar concentrations in vitro and achieved tumor cell regression in vivo without hypercalcemic side effects.^(^ ^18^, ^43^, ^44^, ^45^, ^46^, ^47^ ^)^ Using cells derived from VDR knockout (VDRKO) mice, the VDR was shown to be necessary for mediating growth inhibition by several of these “low calcemic” vitamin D analogs, although some induced non‐specific cytotoxicity in VDRKO cells at high (micromolar) concentrations.^(^ ^48^, ^49^ ^)^ The ability of synthetic VDR agonists to induce antitumor effects in the absence of hypercalcemia has been attributed to differences in binding to DBP, the CYP enzymes, and/or the VDR itself, leading to differences in transport, metabolism, and VDR structure/function compared with the natural ligand 1,25D.^(^ ^50^ ^)^ A caveat is that these characteristics have been measured in vitro and little is known about metabolism, accumulation, or activity of natural or synthetic vitamin D compounds in tumors in vivo. However, xenografts derived from VDRKO mammary tumor cells failed to respond to vitamin D analog therapy, indicating that VDR activation is required in the grafted tumor cells for tumor regression (as opposed to host‐derived fibroblasts, adipocytes, endothelial cells, or immune cells that express VDR).^(^ ^51^ ^)^ In addition, manipulation of either CYP27B1 or CYP24A1 has been shown to alter breast tumor growth in animal models, supporting the idea that cancer cell metabolism of 25D is biologically relevant.^(^ ^52^, ^53^ ^)^

## Clinical Relevance of Vitamin D Pathway Expression in Human Breast Tumors

### VDR during mammary cell lineage determination

**Fig. 1:**
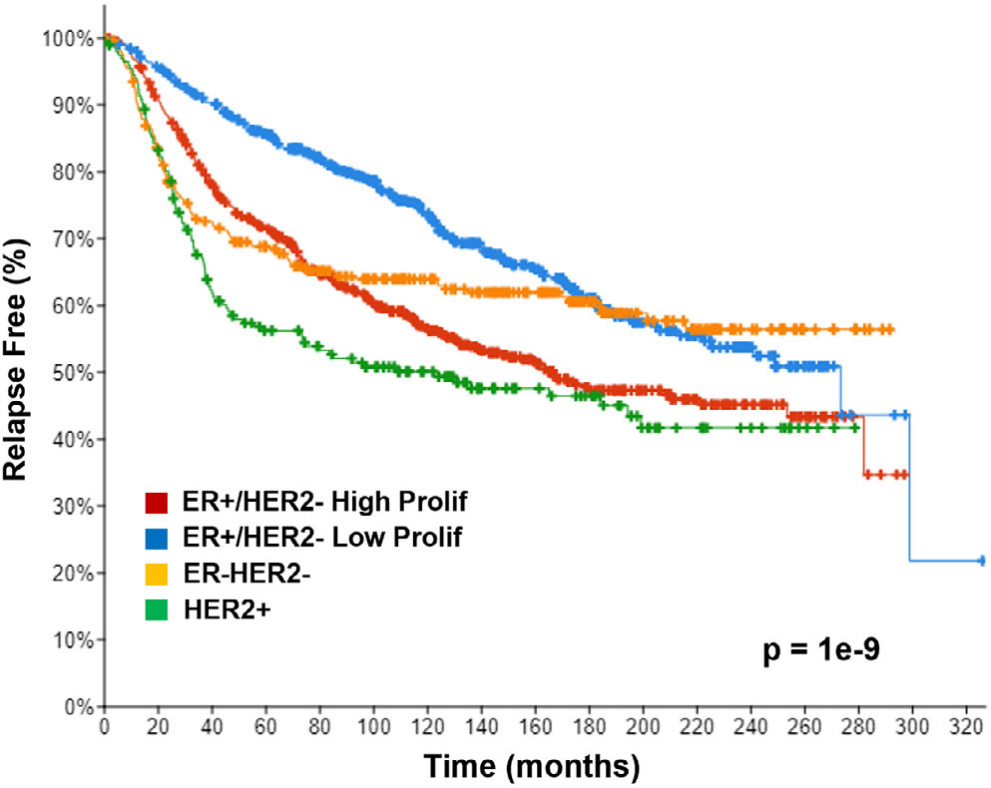
Relapse‐free survival of breast cancer patients stratified by three‐gene classification. Relapse‐free survival of patients over 20 years based on expression of ER, HER2, and Ki67 (proliferation marker) shown as a Kaplan–Meier curve. Cases were derived from the METABRIC data set on cBIO Portal: ER^+^/HER2^–^/High (*n* = 617); ER^+^/HER2^−^/Low (*n* = 640); ER^–^/HER2^–^ (*n* = 309); HER2^+^ (*n* = 198). Relapse included cases where there was loco‐regional relapse, distant relapse, or disease‐specific death.

Both luminal and basal mammary epithelial cells arise from a common stem cell pool, which gives rise to mammary progenitor cells, an early multipotent population that has been demonstrated to express VDR and CYP24A1.^(^ ^54^, ^55^, ^56^ ^)^ Both basal and luminal cell populations originate from these VDR‐positive progenitor cells; however, in the course of basal cell differentiation, VDR expression is silenced. During luminal cell differentiation, both VDR‐positive and VDR‐negative cells arise. Any of these luminal or basal cell populations can theoretically give rise to breast cancer, leading to extraordinary heterogeneity in VDR expression in the normal breast (for further discussion, see Welsh^(^ ^57^ ^)^). Although the population‐specific functional consequences of VDR expression during lineage determination have yet to be clarified, this heterogeneity induces considerable complexity in assessing the potential contribution of the vitamin D pathway to patient outcomes, as each subtype of breast cancer exhibits distinct molecular characteristics and clinical behavior. Whole genome profiling has classified breast cancers into six intrinsic subtypes: luminal A, luminal B, HER2‐enriched, basal‐like, normal breast, and claudin‐low, although these classifications are not yet routine clinical practice.^(^ ^58^, ^59^, ^60^ ^)^ A three‐gene classifier, based on immunohistochemical detection of estrogen receptor α (ER), HER2, and proliferation marker Ki67, is more often used to predict prognosis.^(^ ^61^ ^)^ As shown in Fig. 1, patients whose tumors are ER^+^ with low proliferation rate have the most favorable outcome, whereas those with high proliferation rate and those that lack ER or have amplification of HER2 have less favorable outcomes. As discussed below, VDR expression can be assessed in relation to these subtypes using publicly available data sets.

### VDR expression in relation to clinical parameters

As noted above, the expression of VDR in established human breast cancers suggests that its activation with natural or synthetic ligands might alter the course of the disease. Conversely, patients whose tumors exhibit low VDR expression might be expected to experience poorer prognosis because of the loss of vitamin D's inhibitory actions. Many small studies have evaluated whether VDR protein expression (assessed by immunohistochemistry) in human breast tumors correlates with disease status. As discussed in the recent review by Voutsadakis,^(3^ ^)^ these studies have differed substantially in the patient populations studied, the clinical outcomes assessed, and the specific antibody techniques utilized. Thus, it is not surprising that the data are inconsistent. However, one conclusion that can be made is that the majority of breast cancers express VDR protein at a moderate level, and a significant percentage show high VDR immunoreactivity. A recent meta‐analysis of the published immunohistochemical data has supported the concept that higher VDR protein is beneficial for patient survival,^(^ ^62^ ^)^ but this likely depends on disease stage/grade and subtype.

**Fig. 2:**
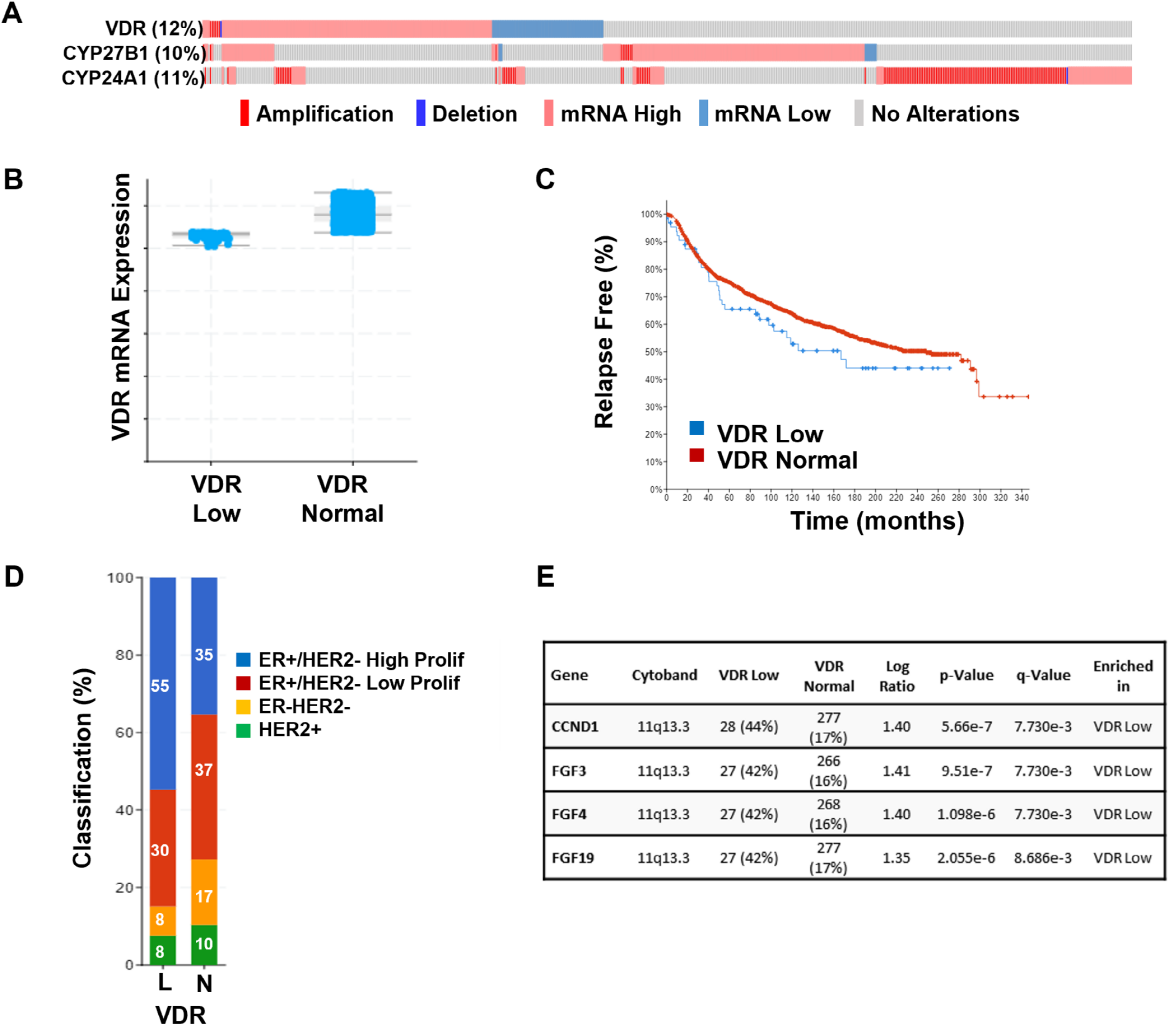
Vitamin D pathway gene expression in the Metabric data set of 1904 breast tumors. (*A*) This oncoprint reports cases in which the indicated alterations (amplification, deep deletion, mRNA upregulation, or mRNA downregulation) in VDR were detected in individual tumor samples (indicated by vertical lines). mRNA changes are based on *Z*‐scores (Illumina Human v3 microarray) and report samples relative to the expression distribution of the VDR gene in tumors that are diploid for the gene of interest (a cut‐off of 1.5 was used). The TGCA data set utilized was the Breast Cancer (METABRIC) consisting of 1904 patients for which complete data were available. Data analysis was conducted within the cBIOPortal for Cancer Genomics at http://www.cbioportal.org/. (*B*) Tumors were categorized as VDR Low or VDR Normal based on Illumina Human v3 microarray data. (*C*) Relapse‐free survival is shown in Kaplan–Meier curve. Relapse included cases where there was loco‐regional relapse, distant relapse, or disease‐specific death. (*D*) Percentage of VDR Low (L) and VDR Normal (N) tumors in each of four histological subtypes of breast cancer. VDR Low tumors were significantly enriched in the ER^+^/HER2^–^/High Proliferation subgroup. (*E*) Copy number alterations in growth‐promoting genes on Chr 11 were enriched in VDR Low tumors.

The availability of genetic data in projects such as ENCODE and large tumor data sets including The Cancer Genome Atlas (TCGA) has facilitated annotation of the VDR gene in human breast cancers in the context of clinical outcomes including survival. TCGA data indicate that breast cancers rarely display mutations or altered levels of expression of the VDR gene. As shown in Fig. 2 *A*, only 12% of tumors in the METABRIC data set of 1904 patient samples^(^ ^63^, ^64^ ^)^ showed abnormalities of the VDR gene (including mutations, amplifications, deletions, and up or down mRNA expression). Contrary to what might be expected based on in vitro studies, human breast tumors with both high and low VDR expression were present in this population, making it difficult to interpret the impact of VDR expression on survival using the entire data set. Stratification of patients by VDR mRNA expression into VDR Low (*n* = 64), VDR High (*n* = 155), and VDR Normal (*n* = 1674) subgroups on cBIO Portal allowed us to selectively examine the effect of low or high VDR expression (Fig. 2*B*) on clinical outcomes. As shown in Fig. 2*C*, patients with VDR Low tumors experienced reduced progression‐free survival (recurrence is defined as cases where there was loco‐regional relapse, distant relapse, or disease‐specific death) compared with those with VDR Normal tumors (167 mos versus 249 mos), although this difference is not statistically significant because of the small number of patients in the VDR Low group. Further annotation of the VDR Low tumors revealed that this subgroup is significantly more likely to exhibit the ER^+^/HER2^–^/High Proliferation phenotype and less likely to exhibit the ER^–^/HER2^–^ phenotype (Fig. 2*D*), providing a possible explanation for poorer outcome of the VDR Low cohort. These observations are consistent with a recent report that low VDR protein expression in clinical breast cancer specimens is associated with higher tumor grade and more aggressive tumor types.^(^ ^23^ ^)^ In this data set, tumors from the VDR Low subgroup also exhibited unique genomic alterations, being less likely to contain PI3KCA mutations and more likely to exhibit copy number alterations (CNAs) in CCDN1 (encodes Cyclin D1) and three fibroblast growth factor genes (FGF3, FGF4, FGF19) than the VDR Normal subgroup (Fig. 2*E*). Cyclin D1 is a known oncogene for breast cells,^(^ ^65^ ^)^ but the relevance of the FGF genes in breast cancer biology has not been extensively explored, nor has their regulation by VDR been reported. Intriguingly, CDDN1 and the FGF genes are clustered together on Chr 11, suggesting that amplification of this genomic region may be enriched in VDR Low tumors, although if true, a mechanism for this is not immediately clear.

**Fig. 3:**
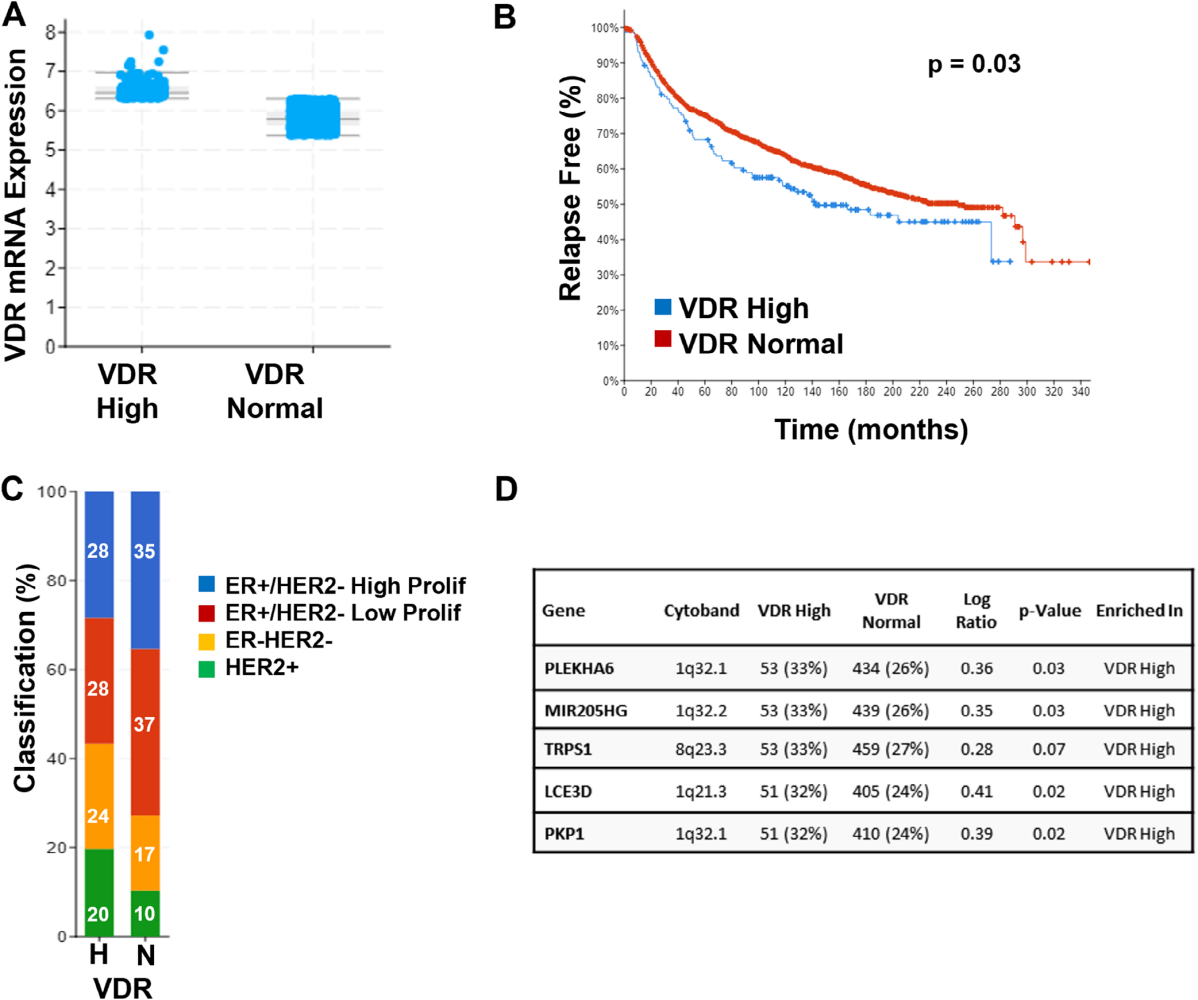
Characteristics of VDR High breast tumors in Metabric data set. (*A*) VDR expression of tumors in data set categorized as High or Normal based on mRNA analysis (Illumina Human v3 microarray). (*B*) Relapse‐free survival of patients whose tumors were categorized as VDR High or VDR Normal is compared in Kaplan–Meier curve. Relapse included cases where there was loco‐regional relapse, distant relapse, or disease‐specific death. (*C*) Percentage of VDR High (H) and VDR Normal (N) tumors in each of four histological subtypes of breast cancer. VDR High tumors were significantly enriched in the ER^–^/HER2^–^ and HER2^+^ subgroups. (*D*) Top five genes with copy number alterations that were significantly enriched in VDR High tumors.

Comparison of VDR High tumors to the VDR Normal subgroup (Fig. 3*A*, *B*) unexpectedly indicated that this subgroup also exhibited reduced regression‐free survival (142 versus 249 mos, *p* = 0.032). Profiling of the VDR High tumors indicated these were more likely to be ER^–^/HER2^–^ or HER2^+^ and were enriched in CNAs on Chr 1 and 8 (Fig. 3*C*, *D*). A mechanistic link, if any, between high VDR expression, CNA enrichments, and survival is not predicted based on the literature and warrants further study. Although speculative, it is possible that the unique CNA enrichments in these tumor subtypes (ER^–^/HER2^–^ and HER2^+^) activate pathways that corrupt VDR signaling sufficiently to abrogate the anticancer effects of 1,25D. Collectively, the TCGA data indicate that VDR High and VDR Low breast tumors are molecularly distinct, although both subsets of patients exhibit poorer survival than patients whose tumors express VDR in the normal range. The underlying basis for these differences remain to be resolved but does not appear to be related to alterations (ie, amplifications or deletions) in Chr 12 where the VDR gene resides.

Linking VDR gene expression to clinical outcomes is further complicated by alternative splicing, which may yield up to 14 distinct VDR transcripts.^(^ ^66^ ^)^ In breast, four different transcripts have been identified—two of which are predicted to produce full‐length VDR and two predicted to produce truncated VDR proteins of 395 or 36 amino acids. Marik and colleagues^(^ ^67^ ^)^ reported that the level and pattern of VDR splice variants was markedly different in tissue derived from breast cancer compared with normal breast. Breast cancer tissues showed extensive heterogeneity and variability (particularly in the shorter variants, which are barely detectable in normal tissue) and expressed markedly lower levels of full‐length VDR transcripts. Further studies to determine whether VDR transcript variants are clinically relevant in breast and other cancers are warranted. In summary, although low VDR expression in breast tumors may correlate with reduced survival, high VDR expression does not associate with better survival. Categorization of VDR expression (either mRNA or protein) in breast cancer in relation to clinical parameters remains complicated because of the heterogeneity of breast cancer with its high frequency of genomic instability.

### CYP27B1 expression in breast cancers

Few studies have addressed CYP27B1 expression in relation to clinical outcomes in breast cancer, although as noted above its expression has been shown to confer growth inhibition in response to 25D (the major circulating metabolite) in vitro and in mouse models.^(^ ^4^, ^68^, ^69^ ^)^ Using the same approaches discussed for VDR, we found that 10% of breast cancers in the METABRIC data set displayed alterations at the CYP27B1 locus, with >95% of these being amplifications or mRNA upregulations. There was no evidence that loss of CYP27B1 was common in breast cancer, and survival was not improved in patients whose tumors had increased expression of CYP27B1.

### CYP24A1 amplification in breast cancers

**Fig. 4:**
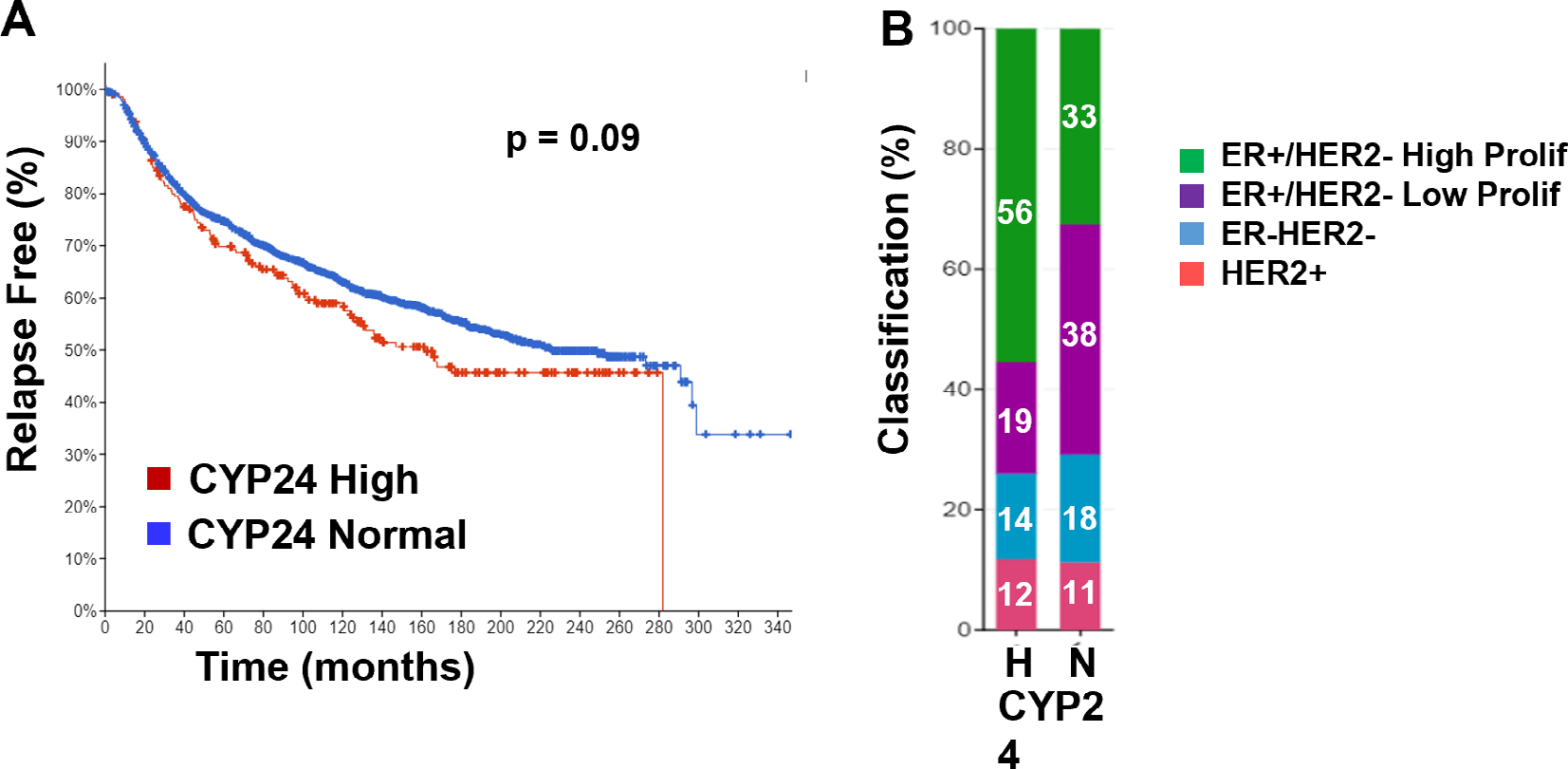
CYP24A1 expression and clinical outcomes in the Metabric data set of 1904 breast tumors. (*A*) Relapse‐free survival of patients whose tumors were categorized with CYP24A1 amplifications and/or upregulation (CYP24 High) versus those with expression in the normal range (CYP24 Normal) based (Illumina Human v3 microarray). Relapse included cases where there was loco‐regional relapse, distant relapse, or disease‐specific death. (*B*) Percentage of CYP24 High and CYP24 Normal tumors in each of four histological subtypes of breast cancer. CYP24 High (H) tumors were significantly enriched in the ER^+^/HER2^–^/High Proliferation subgroup compared to CYP24 Normal (N) tumors.

Up to 10% of human breast cancers have been demonstrated to exhibit a ~1 Mb region of recurrent amplification at 20q13. The presence of this large amplified genomic region (which may contain more than 300 distinct genes) is a known oncogenic event that is associated with poor prognosis. The gene that encodes CYP24A1 lies in this region, leading to the hypothesis that reduced ligand availability to VDR due to excessive catabolism might limit VDR activation and contribute to disease progression in these tumors. Indeed, 8% of tumors in the METABRIC data set of 1904 patients exhibit CYP24A1 amplification and another 3% had high levels of CYP24A1 mRNA in the absence of amplification (Fig. 2*A*); relapse‐free survival was significantly reduced in the subgroup with High CYP24A1 expression (Fig. 4*A*). Tumors with high expression of CYP24A1 were more likely to be ER^+^ with a high proliferative index compared with those with low CYP24A1 expression (Fig. 4*B*). However, because this amplification is complex, with multiple subregions and many genes co‐amplified with CYP24A1, it is difficult to determine the extent to which increased CYP24A1 expression contributes to disease progression.^(^ ^53^, ^70^ ^)^ Although cellular studies have demonstrated oncogenic properties of CYP24A1 in vitro,^(^ ^53^ ^)^ expression of CYP24A1 protein in human breast cancers is not consistently elevated.

### Other factors affecting vitamin D pathway functionality in breast cancers

Based on these genomic data, expression of the genes that encode VDR, CYP27B1, and CYP24A1 appears to be in the normal range for the vast majority of breast tumors. Furthermore, the changes that are detected are predominantly mutually exclusive (Fig. 2*A*). Thus, the extent to which deregulation of the vitamin D endocrine system in breast cancer induces pathological consequences with respect to disease development or progression remains unclear. It should be emphasized that demonstration of VDR expression at the mRNA or protein level in tumors does not necessarily indicate that the vitamin D pathway is functional. Clearly, VDR activation in tumors requires ligand, which depends on the patient's overall vitamin D status as well as the relative activity of CYP27B1 and CYP24A1 within individual tumor cells. Few studies that have examined the clinical impact of tumor VDR expression have included assessment of patient vitamin D status. Serum 25D is often suboptimal in breast cancer patients,^(^ ^71^, ^72^ ^)^ but there are no published data on tumor accumulation of vitamin D metabolites in relation to serum concentrations. Thus, the serum 25D concentration required to optimally activate VDR in tumor cells is unknown.

In addition to ligand availability, cellular factors activated during carcinogenesis may corrupt VDR function even in the absence of altered expression. For example, oncogenes such as ras and EMT promoting transcription factors disrupt VDR signaling, leading to desensitization to 1,25D‐mediated actions.^(^ ^8^, ^73^ ^)^ In addition, the VDR gene is subject to silencing via methylation^(^ ^67^ ^)^ which was demonstrated by comparison of eight human breast cancers and seven adjacent normal breast samples. Methylation‐specific PCR confirmed highly methylated CpG islands (40% to 65%) in primary breast tumors but low levels in adjacent normal breast tissue (5% to 15%). In vitro, treatment of breast cancer cell lines with demethylating agents coordinately enhanced the expression of VDR and sensitivity to 1,25D‐mediated growth inhibition.

Other factors that may impede vitamin D's antitumor effects include phosphorylation of VDR itself or its transcriptional partners, altered VDR subcellular localization, disruption of target genes rendering them non‐functional or non‐responsive to VDR regulation, etc.^(^ ^74^, ^75^, ^76^ ^)^ Collectively, these observations demonstrate multiple mechanisms by which vitamin D signaling can be corrupted in cancers and are consistent with the demonstration that explants from breast cancers are less sensitive to 1,25D than explants from adjacent normal tissue or healthy breast epithelium.^(^ ^77^ ^)^ Clarification of the physiologic, cellular, and molecular determinants of sensitivity to 1,25D‐mediated anticancer actions remains a critical gap in translating preclinical findings into prevention/treatment strategies and population guidelines. Thus, more detailed characterization of the vitamin D signaling pathway in both experimental models and clinical specimens is warranted.

## Impact of Vitamin D Signaling in Breast Cancer Prevention and Treatment: Animal Models

The demonstration that normal breast cells also express VDR, CYP27B1, and CYP24A1 indicates that dietary vitamin D status might impact glandular function during puberty, pregnancy, or lactation and/or alter the risk for breast cancer development. Mice with targeted deletion of vitamin D pathway genes have been studied in relation to normal development as well as in spontaneous, induced, and transgenic models of breast cancer. Mice bearing xenografts of human breast tumors have been tested for sensitivity to dietary vitamin D modifications and VDR agonist treatments. Data from these animal models are summarized in Table 1, {TBL 1} and a few will be highlighted here. Ablation of VDR (in the setting of normocalcemia) altered mammary gland development during the rapid expansion associated with both puberty and pregnancy. Glands from VDRKO mice exhibited increased proliferation and decreased apoptosis both in vivo during puberty and pregnancy and when studied as explants in the presence of estrogen and progestins.^(^ ^78^, ^79^ ^)^ Tissue‐specific deletion approaches indicated that VDR functioned in both epithelial and stromal compartments of the breast during puberty.^(^ ^80^ ^)^ These data imply that VDR functions to fine‐tune hormone‐stimulated proliferation in normal breast cells. This concept is supported by the demonstration that mice with mammary‐specific deletion of CYP24A1 (which would be predicted to increase cellular accumulation of 1,25D and VDR activity) exhibited reduced numbers of terminal end buds, stunted ductal outgrowth, and less branching during puberty, as well as delayed formation of alveoli during pregnancy compared with wild‐type (WT) mice.^(^ ^81^ ^)^ Despite this evidence that VDR contributes to breast cell turnover, VDRKO mice did not develop spontaneous tumors with age, likely because of a shortened life span associated with adipose atrophy and debilitating epidermal lesions.^(^ ^78^, ^82^ ^)^ However, a recent study reported that CYP27KO mice developed spontaneous tumors (including some breast carcinomas) with age via mechanisms involving enhanced oxidative stress.^(^ ^83^ ^)^

**Table 1:** Actions of Vitamin D and the Vitamin D Receptor (VDR) in Selected Animal Models of Breast Cancer

Model	Study description	Outcome
Spontaneous lesions: Development, preneoplasia, tumorigenesis	VDR knockout (VDRKO) mice maintained on rescue diet—mammary development and aging studies^(^ ^71^, ^72^, ^75^ ^)^	High VDR expression in differentiated epithelial cells. Increased hormone‐stimulated proliferation and branching in VDRKO glands in organ culture and in vivo compared with wild‐type (WT) mice. Delayed glandular regression after lactation in VDRKO relative to WT mice.
	Mammary epithelial‐ or adipocyte‐specific VDRKO—development study^(^ ^73^ ^)^	VDR in both adipose and epithelial cells functions to restrict pubertal glandular proliferation/development. Epithelial VDR (but not adipose VDR) functions to restrict alveologenesis during pregnancy. 1,25D induced secretion of IL‐6 and leptin via adipose VDR ex vivo.
	CYP27B1KO rescue diet and 1,25D treatment—aging study^(^ ^76^ ^)^	Increased age‐related spontaneous tumor burden in CYP27B1KO mice compared with WT mice. Prevented by either 1,25D or antioxidants, implying that lack of 1,25D enhanced oxidative stress and DNA damage. Mechanistic data implicated HGF and MET receptor in driving tumorigenesis.
	Mammary epithelium‐specific deletion of CYP24A1 KO—development study^(^ ^74^ ^)^	CYP24A1 deletion in mammary epithelium reduced proliferation and inhibited ductal budding, outgrowth and branching (at puberty), and alveologenesis (in early pregnancy).
MMTV‐Ron mice: Metastatic mammary tumors develop in response to Ron oncogene expression.^(77^ ^)^	MMTV‐Ron mice were crossed with VDRKO mice. Hyperplasia, tumor burden, and β‐catenin signaling were evaluated.	Enhanced Ron‐mediated mammary hyperplasia, tumor burden, and metastasis to lungs and liver in VDRKO versus WT mice. VDRKO tumors displayed elevated β‐catenin signaling.
MMTV‐Neu mice: Mammary tumors develop in response to targeted expression of Neu oncogene (models HER2‐positive human breast cancer).	MMTV‐Neu mice were crossed with VDRKO mice. Ductal morphology, preneoplastic lesions, and tumor burden were evaluated.^(^ ^71^ ^)^	High expression of VDR detected in MMTV‐Neu tumors and lung metastatic foci. Abnormal ductal morphology in VDRKO and VDR‐HET mice. Increased tumor incidence in VDR‐HET versus WT mice on MMTV‐Neu background.
	MMTV‐Neu mice were treated with VDR agonist BXL0124.^(98^ ^)^	BXL0124 decreased tumor weight, incidence, and multiplicity and inhibited ErbB2, Erk, and Akt signaling.
	MMTV‐Neu mice were treated with BXL0124 ± CDDO‐Im (synthetic triterpenoid) either before or after tumor onset.^(^ ^47^ ^)^	In prevention protocol, both BXL0124 and CDDO‐Im delayed tumor development, but the combination was most effective. In the therapeutic protocol, administration of the combination did not reduce tumor burden.
bLHβ‐CTP mice: Mammary hyperplasia and spontaneous tumors develop in response to chronic, systemic LH production.	Effect of short‐term treatment of tumor‐bearing mice with EB1089 on proliferation and tumor burden.^(^ ^99^ ^)^	LH‐driven tumors had high VDR expression. EB1089 inhibited tumor cell proliferation and reduced tumor burden in ~50% of treated mice.
MMTV‐PyMT mice: Rapid‐onset mammary tumors that metastasize to lung. Develop in response to targeted expression of polyoma middle T antigen.	Tumorigenesis was evaluated in MMTV‐PyMT mice fed low (25 IU/kg) versus standard (1000 IU/kg) vitamin D diets and in mice perfused with 25D or 1,25D. Tumor vitamin D metabolites were measured.^(^ ^63^ ^)^	Low dietary vitamin D accelerated tumorigenesis relative to standard diet. Systemic perfusion with 25D or 1,25D delayed tumorigenesis and decreased lung metastasis. Both 25D and 1,25D were detected in tumors.
	Lung metastasis was evaluated in MMTV‐PyMT mice fed low (25 IU/kg) versus standard (1000 IU/kg) vitamin D diets. Tumor cells were studied ex vivo.^(^ ^21^ ^)^	Vitamin D deficiency enhanced lung metastasis in vivo and markers of epithelial‐mesenchymal transition (EMT) in vitro. Mechanisms identified included co‐localization of chemokine CXCL12 and its receptor CXCR4 in the lung metastatic niche and increased expression of pSTAT3 and ZEB1 (EMT drivers).
	Tumor development was evaluated in MMTV‐PyMT mice with mammary‐specific deletion of CYP27B1.^(^ ^62^ ^)^	Targeted ablation of CYP27B1 in MMTV‐PyMT mice accelerated mammary hyperplasia and tumorigenesis. NfKB and JAK–STAT signaling were increased in CYP27B1 ablated tumors. CYP27B1 ablation reduced tumor 1,25D level.
Chemically induced mammary tumors: Mammary glands primed with MPA (progesterone analog) and injected with DMBA (dimethylbenzanthracene) develop mammary tumors that express estrogen receptor (ER) and progesterone receptor (PR).	Glandular morphology and mammary tumorigenesis was studied in WT and VDRKO mice fed high‐calcium rescue diet.^(^ ^71^ ^)^	Total tumor incidence was similar in WT and VDRKO mice, but VDRKO tumors were predominantly negative for ER and PR and exhibited transdifferentiation toward epidermis and hair. Glands from VDRKO mice showed impaired proliferative response to MPA stimulation compared with WT mice. Tumor histology in VDRKO mice was suggestive of *wnt* pathway activation.
	Tumor incidence and burden were evaluated in mice fed diets containing standard (1000 IU/kg) or supplemental (20,000 IU/kg) levels of vitamin D_3_ before DMBA treatment.^(^ ^31^ ^)^	Supplemental dietary vitamin D_3_ reduced tumor incidence and burden, inhibited pro‐survival autophagy markers, and increased accumulation of p62. Data supported reduction in tumor autophagy with vitamin D_3_ supplementation.
Xenograft models: Human breast cancer cells injected into immunodeficient mice at various sites to mimic primary tumor progression and/or metastatic colonization.	Comparison of orthotopic tumors derived from MDA‐MB‐231 cells expressing control or CYP24A1‐targeted shRNA.^(^ ^53^ ^)^	Suppression of CYP24A1 in MDA‐MB‐231 tumors reduced tumor weight and expression of Ki67 (proliferation marker) and CD37 (microvessel marker) while enhancing apoptosis and necrosis. Data also report gene expression profiles in MCF‐7 and MDA‐MB‐231 cells upon CYP24A1 silencing in vitro.
	Comparison of skeletal metastases after intracardiac injection of MDA‐MB‐231 cells expressing control or VDR targeted shRNA.^(^ ^23^ ^)^	Ablation of VDR in injected tumor cells promoted EMT, cancer cell mobility (migration), and invasiveness, thereby facilitating skeletal colonization.
	Combination therapy of vitamin D analogs and an aromatase inhibitor was evaluated in MCF‐7 xenografts.^(^ ^43^ ^)^	PRI‐2191 or PRI‐2205 (non‐calcemic vitamin D analogs) potentiated the antitumor effects of the aromatase inhibitor anastrazole in MCF‐7 tumor‐bearing mice. The combination treatment reduced aromatase gene expression and activity and downregulated ER expression.

The potential role of VDR signaling in breast cancer prevention has been further tested in transgenic and chemically induced mouse models. Global VDR heterozygous mice were more sensitive to tumorigenesis than WT mice in the MMTV‐Neu transgenic model of breast cancer, which models HER2^+^ human breast cancer.^(^ ^78^ ^)^ VDR ablation also enhanced tumorigenesis driven by the chemical carcinogen DMBA and the RON oncogene, whereas CYP27B1 ablation enhanced tumorigenesis driven by the polyoma middle T antigen (MMTV‐PyMT model).^(^ ^68^, ^84^, ^85^ ^)^ Conversely, administration of VDR agonists (either through dietary vitamin D manipulation or treatment with vitamin D metabolites or analogs) has been shown to delay tumorigenesis in many of these same models (Table 1). Pathways implicated in the tumor‐preventive effects of VDR include WNT/β‐Catenin, ERBB2/ERK/AKT, JAK/STAT, NfκB, and ERα, all of which are oncogenic and are suppressed by vitamin D signaling. Overall, these studies support the concept that the vitamin D pathway is a physiologically relevant modulator of mammary gland development whose deregulation induces changes consistent with enhanced susceptibility to carcinogenesis.

Translation of these animal studies to prevention or treatment of human breast cancer via targeting the vitamin D pathway has yet to be fulfilled. It is clear that vitamin D deficiency is common in breast cancer patients at diagnosis.^(^ ^86^ ^)^ Estebanez and colleagues conducted a meta‐analysis of 68 studies that assessed vitamin D intake or serum levels of 25D or 1,25D in relation to breast cancer risk. The data indicated a significant protective effect between 25D (but not 1,25D or dietary D) and breast cancer in both cohort studies and case‐control studies. Subgroup analysis suggested that the association between 25D and risk was only significant in premenopausal women. Another recent meta‐analysis of 12 observational studies with >8500 subjects found a significant reduction in overall survival, breast cancer–specific survival, and disease‐free survival in breast cancer patients with low serum 25D.^(^ ^87^ ^)^ The only large randomized controlled trial of vitamin D supplementation specifically designed to examine cancer risk was the VITAL study (25,871 men ≥50 years and women ≥55 years). Although initial analysis indicated that daily supplementation with 2000 IU vitamin D3 for 5 years did not significantly impact cancer incidence,^(88^ ^)^ secondary analysis reported a significant reduction in advanced cancers (metastatic or fatal) for those randomized to vitamin D compared with placebo.^(^ ^89^ ^)^ The protective effect of vitamin D supplementation in the VITAL study appeared to be restricted to those participants with normal body mass index (BMI), consistent with the known adverse effects of obesity on vitamin D action.^(^ ^90^ ^)^ Despite the large number of participants recruited to this trial, there were insufficient numbers of breast cancer cases to specifically test the impact of vitamin D_3_ supplementation on breast cancer incidence or mortality. A limitation of the VITAL trial was that serum 25D was not measured, therefore subjects could not be stratified by vitamin D status at baseline or after supplementation. Other limitations common to randomized control trials of vitamin D supplementation that may have affected outcomes in VITAL have recently been reviewed.^(^ ^91^ ^)^

## Genomic Profiles of VDR Signaling

Several studies have profiled the whole‐genome effects of 1,25D in breast cancer model systems including non‐transformed cell lines (hTERT‐HME1, HME, MCF10A), human breast cancer cell lines representative of ER^+^ (MCF7), HER2^+^ (SKBR3), and Triple Negative (MDA‐MB‐231, SUM159, Hs578T), and patient explants. As might be predicted given the heterogeneous nature of these cell lines, which were derived from different subtypes of breast cancer, only a small degree of overlap in the genes altered in response to VDR agonists was noted. For example, two studies that profiled gene expression in response to 1,25D treatment of breast cancer explants in culture, which were primarily ER^+^ ductal carcinomas, revealed only four commonly regulated genes (CYP24A1, CLMN, EFTUD1, SERPINB1). The fact that these genes were also found to be regulated by 1,25D in cell culture models^(^ ^36^ ^)^ suggested they may represent “signature” VDR targets in breast cancer. Interestingly, there were no genes that were commonly downregulated by 1,25D in all model systems. This observation is consistent with the known heterogeneity of gene repression by VDR, which is not typically associated with binding to canonical vitamin D response elements and instead is mediated through cell‐specific co‐repressors.^(^ ^92^ ^)^

The four 1,25D genes commonly upregulated in breast cancer cell lines, as well as KLK6 (kallikrein‐related peptidase 6)—a gene that was highly induced in both tumor explants and MCF7 cells—were confirmed as 1,25D regulated in breast cancer cell lines and in a subset of human clinical samples from normal tissue and breast cancer. Observational studies have indicated that high expression of KLK6, CLMN (encodes Calmin, a membrane calponin‐like protein), and EFTUD1 (encodes elongation factor like GTPase 1, a ribosome biogenesis factor) in breast tumors promotes better survival, supporting a link between vitamin D signaling and disease outcomes.^(^ ^77^ ^)^ SERPINB1 encodes Serpin Family B Member 1 (a proteinase that primarily functions in an anti‐inflammatory capacity), but its role in breast cancer has not been well studied.

Direct comparison of the effect of 1,25D on six putative VDR target genes (CYP24A1, ITGβ3, SLC1A1, KDR, BIRC3, and GLUL)^(^ ^37^ ^)^ in various breast cell lines indicated that, with the exception of CYP24A1, which was induced in all cell lines, breast cancer cell lines were less responsive to 1,25D than were immortalized cells with respect to induction or repression of the selected genes, despite reasonably comparable VDR expression. As noted above, there are many variables that can directly alter VDR function, and the presence of genomic alterations in cancer cells could indirectly deregulate target gene responses to 1,25D or downstream pathways (such as proliferation or apoptosis).

Another source of heterogeneity involves VDR interaction with other nuclear receptors as well as transcriptional co‐activators and co‐repressors. VDR is a member of the NR1I subfamily of nuclear hormone receptors, which also includes the pregnane X receptor (PXR; NR1I2) and the constitutive androstane receptor (CAR; NR1I3). These receptors and others (such as the retinoic acid receptor [RAR] isoforms and the peroxisome proliferator‐activated receptor [PPAR] isoforms) dimerize with the α, β, or γ subtypes of retinoid X receptors (RXRs, NR2B1–3) to effect cell‐specific programs of gene transcription in response to their respective lipophilic ligands. Despite the known requirement of RXRs as dimerization partners for VDR and the possibility that activation of related nuclear receptors might compete with VDR for RXR binding,^(^ ^93^ ^)^ few studies have accounted for the impact of other lipophilic ligands on 1,25D‐mediated gene expression profiles. Based on available data, all of which have been generated in vitro, there is no “tumor gene signature” that is generally reflective of vitamin D exposure in breast cancer patients. This research gap should be addressed using both in vitro and in vivo approaches. More sophisticated techniques such as single‐cell sequencing, as was recently reported for vitamin D analog treatment in a mouse model of prostate cancer,^(94^ ^)^ will be useful to comprehensively assess vitamin D signaling in heterogeneous breast cancer tissue.

## Cellular Pathways Targeted by VDR: EMT, Stem Cells, and Tumor Microenvironment

### Vitamin D signaling and EMT

An early event in mammary carcinogenesis is the acquisition of a mesenchymal phenotype by luminal or basal epithelial cells in the ducts or lobules. This epithelial‐mesenchymal transition (EMT) is characterized by changes in morphology, migratory ability, and gene expression (loss of E‐cadherin, gain of vimentin) and is driven by numerous factors including TGFβ, TWIST, SNAIL, SLUG, JAK–STAT, and ZEB1. In cellular models of EMT, VDR expression is reduced 70% to 90% depending on the specific inducer used,^(22^ ^)^ and depletion of VDR has been shown to promote EMT.^(^ ^23^ ^)^ Despite low VDR expression, cells that have undergone EMT retained sensitivity to 1,25D‐mediated gene expression. In vitro, 1,25D partially reversed the EMT phenotype (decreased invasion/motility) as evidenced by reduction in pSTAT3, ZEB1, and vimentin and increase in E‐cadherin.^(^ ^21^, ^95^, ^96^, ^97^, ^98^ ^)^ Furthermore, dietary vitamin D inhibited EMT and lung metastasis via reduction in ZEB1 and STAT3 expression in the MMTV‐PyMT model of breast cancer.^(^ ^21^ ^)^ In a xenograft model of human breast cancer, depletion of VDR promoted EMT and enhanced skeletal colonization.^(^ ^23^ ^)^

### Vitamin D signaling and stem cells

One of the consequences of EMT is the emergence of cells that express markers and properties of mammary progenitor (stem) cells. Breast cancers are thought to arise from transformed mammary progenitor cells, which are identified by surface markers (CD44^HI^/CD24^LOW^) and/or activity of aldehyde dehydrogenase 1 (ALDH1^+^) and can be propagated in vitro as nonadherent spheres (“mammospheres”). Pervin and colleagues^(^ ^99^ ^)^ used various breast cancer cell lines to demonstrate that VDR protein was reduced in mammospheres compared with monolayer cultures. Furthermore, they reported that VDR overexpression reduced, whereas VDR knockdown enhanced, mammosphere number. In another study of human triple negative breast cancer cells, Thakkar and colleagues^(^ ^100^ ^)^ demonstrated that 1,25D inhibited mammosphere formation, decreased the percentage of ALDH^+^ cells, and reduced CSC‐associated markers, including CD44. More recently, synthetic VDR agonists have been shown to block mammosphere formation and stem cell markers in a model of early‐stage basal‐like breast cancer (MCF10DCIS.com cells) via downregulation of pathways (such as NOTCH and CD44) that are required for maintenance of breast cancer stem‐like cells.^(^ ^42^, ^101^, ^102^ ^)^ Importantly, treatment of tumor‐bearing mice with VDR agonists led to reduction in CD44 signaling in vivo.^(^ ^101^ ^)^ Inhibitory effects of 1,25D on stem cell populations have also been demonstrated in a WNT1‐driven murine mammary tumor model.^(^ ^103^ ^)^ Gene expression profiling identified specific pathways altered by vitamin D, implicating HES/NOTCH/MYC and WNT/β‐catenin pathways in regulation of stemness.^(^ ^42^ ^)^ In summary, these studies highlight the existence of functional VDR in breast cancer stem‐like cells that, when activated, induced gene expression programs that inhibited stem cell function. Although the clinical relevance of these observations has yet to be validated, the work adds new insight into the actions of vitamin D in breast cancer and provides fertile ground for additional research.

### Vitamin D signaling and the tumor microenvironment

The tumor microenvironment (TME) refers to all components within tumors other than the cancerous cells, and includes other cell types that infiltrate the tumor (immune cells, endothelial cells, fibroblasts) as well as factors (proteins, carbohydrates, extracellular vesicles, nucleic acids, etc.) secreted by all of these cell types. Vitamin D signaling has been shown to affect most cell types in the TME, including cancer‐associated fibroblasts, tumor‐infiltrating lymphocytes, and endothelial cells, leading to beneficial effects such as inhibition of tumor progression, blockade of tumor angiogenesis, and enhancement of antitumor immunity.^(^ ^104^ ^)^ Hyaluronic acid (HA), a high molecular weight linear glycan that serves as the best characterized ligand of the stem cell marker CD44, has been identified as a target of VDR that is secreted by tumor cells and accumulates in the TME.^(^ ^22^, ^26^ ^)^ CD44 is a cell surface glycoprotein that drives STAT3 signaling and stem cell survival when activated by HA. There is overwhelming evidence that excessive HA in the tumor microenvironment promotes aggressiveness^(^ ^105^ ^)^ and TCGA data support the concept that high expression of the major HA synthesizing enzyme HAS2 is associated with reduced patient survival. 1,25D markedly suppressed HAS2 and reduced HA secretion by breast cancer cells, and the growth inhibitory effects of 1,25D3 were partially reversed in the presence of exogenous HA.^(^ ^22^ ^)^ HAS2 expression and HA production are elevated in models of mammary EMT, indicating that deregulation of HA production occurs early in breast tumorigenesis and is associated with the emergence of CD44^+^ stem cells. 1,25D blocked HA production during EMT and acted synergistically with an HA inhibitor to suppress cell growth. Furthermore, analysis of mammary gland and tumors from VDRKO mice demonstrated that loss of VDR was associated with enhanced HAS2 and HA accumulation in vivo.^(^ ^22^ ^)^ Thus, vitamin D signaling directly compromises breast cancer stem cell viability and potentially delays tumor progression via interruption of HA‐CD44 survival signaling in the TME. For more detailed discussion of these and other effects of the vitamin D pathway on the TME, see the review by Wu and colleagues.^(^ ^104^ ^)^

## Summary

Breast cancers are highly heterogeneous, yet many express VDR, suggesting that vitamin D status may be clinically relevant for women living with this disease. Mechanistic studies have demonstrated that vitamin D signaling opposes multiple proliferative pathways in both normal breast tissue and in breast cancers, including those driven by reproductive hormones. Recent studies in model systems have highlighted inhibitory effects of vitamin D signaling on EMT and breast cancer stem cells, which would be predicted to reduce their capacity to drive metastasis, drug resistance, and poor survival. These effects include inhibition of CD44 signaling via disruption of HAS2 production of its ligand HA. In addition, vitamin D has the potential to modulate various non‐cancerous cell types in the tumor microenvironment to promote tumor immunity and inhibit tumor angiogenesis. Translation of these findings into prevention or treatment of human breast cancer will require attention to several research gaps, including identification of common and relevant VDR targets in distinct breast cancer subtypes, clarification of the mechanisms and importance of accumulation and turnover of vitamin D metabolites in tumors, and discovery of regulatory mechanisms that enhance vitamin D signaling in the TME.

## Conflict of Interest

No conflicts of interests.

### Peer Review

The peer review history for this article is available at https://publons.com/publon/10.1002/jbm4.10582.
